# Omentum-derived matrix enables the study of metastatic ovarian cancer and stromal cell functions in a physiologically relevant environment

**DOI:** 10.1016/j.mbplus.2023.100136

**Published:** 2023-11-22

**Authors:** Lisa J. Neilson, Douglas Cartwright, Maija Risteli, Elina M. Jokinen, Lynn McGarry, Toni Sandvik, Konstantina Nikolatou, Kelly Hodge, Samuel Atkinson, Maria Vias, Emily J. Kay, James D. Brenton, Leo M. Carlin, David M. Bryant, Tuula Salo, Sara Zanivan

**Affiliations:** a Cancer Research UK Scotland Institute, Glasgow, UK; b Research Unit of Population Health, Medical Research Center Oulu, University of Oulu and Oulu University Hospital, Oulu, Finland; c School of Cancer Sciences, University of Glasgow, Glasgow, UK; d Department of Bacteriology and Immunology, Translational Immunology Research Program, University of Helsinki, Finland; e Cancer Research UK Cambridge Institute, University of Cambridge, Li Ka Shing Centre, Robinson Way, Cambridge, UK; f Department of Pathology, University of Helsinki, Helsinki, Finland; g Department of Oral and Maxillofacial Diseases, Clinicum, Faculty of Medicine, University of Helsinki, Helsinki, Finland

**Keywords:** Extracellular matrix, Ovarian cancer, CAF, Matrisome, Omentum

## Abstract

High-grade serous (HGS) ovarian cancer is the most lethal gynaecological disease in the world and metastases is a major cause. The omentum is the preferential metastatic site in HGS ovarian cancer patients and *in vitro* models that recapitulate the original environment of this organ at cellular and molecular level are being developed to study basic mechanisms that underpin this disease. The tumour extracellular matrix (ECM) plays active roles in HGS ovarian cancer pathology and response to therapy. However, most of the current *in vitro* models use matrices of animal origin and that do not recapitulate the complexity of the tumour ECM in patients.

Here, we have developed omentum gel (OmGel), a matrix made from tumour-associated omental tissue of HGS ovarian cancer patients that has unprecedented similarity to the ECM of HGS omental tumours and is simple to prepare. When used in 2D and 3D *in vitro* assays to assess cancer cell functions relevant to metastatic ovarian cancer, OmGel performs as well as or better than the widely use Matrigel and does not induce additional phenotypic changes to ovarian cancer cells. Surprisingly, OmGel promotes pronounced morphological changes in cancer associated fibroblasts (CAFs). These changes were associated with the upregulation of proteins that define subsets of CAFs in tumour patient samples, highlighting the importance of using clinically and physiologically relevant matrices for *in vitro* studies. Hence, OmGel provides a step forward to study the biology of HGS omental metastasis. Metastasis in the omentum are also typical of other cancer types, particularly gastric cancer, implying the relevance of OmGel to study the biology of other highly lethal cancers.

## Highlights

• OmGel is a patient-derived matrix with unprecedented similarity with the matrix of ovarian metastatic tumours.
• OmGel is a physiologically and clinically relevant matrix for functional assays *in vitro*.
• OmGel supports invasion of ovarian cancer cells.
• OmGel modulates CAF phenotype.

## Introduction

High-grade serous (HGS) ovarian cancer is the most common and aggressive ovarian cancer type, and the most lethal gynaecological disease [1], [2]. It is estimated that over 200,000 women died from ovarian cancer in 2020 and this is expected to rise to over 300,000 by 2040 [3]. The major cause of this is its highly metastatic nature and the limited availability of effective therapies to oppose it. The omentum is a highly vascularised visceral depot of adipose tissue layered on the surface of the intra-peritoneal organs, which has immune functions, and which becomes the preferential metastatic site in patients with HGS ovarian cancer [1], [2]. The omentum provides an environment that supports the rapid growth of metastatic tumours and their spread within the peritoneal cavity and adjacent organs [2], [4]. Research aimed at understanding the biology of metastatic tumours in the omentum is therefore essential to find strategies to oppose HGS ovarian cancer metastasis. To this aim, there is the need for *in vitro* models that faithfully recapitulate the microenvironment of HGS omental metastasis in patients.

The extracellular matrix (ECM) is composed of myriad proteins, which all together define what is called “Matrisome” [5], [6]. The Matrisome includes both structural ECM components (core Matrisome) and proteins that interact with or remodel the ECM (Matrisome-associated) [5], [6]. Tumour ECM proteins actively contribute to tumour development, metastatic dissemination and response to therapy by influencing the behaviour of cancer, stromal and immune cells. Highlighting the importance of the tumour ECM in cancer, targeting specific ECM components strongly interferes with tumour formation and progression [7], [8], [9]. In tumours, the composition of the ECM evolves during disease progression and differs at different tumour sites [7], [8], [9]. HGS ovarian omental tumours contain vast amounts of ECM and mass spectrometry (MS) proteomic and transcriptomic analysis of these tumours have determined their ECM composition and how it evolves with disease progression [10], [11]. Notably, these analyses have identified a subset of ECM components that correlate with the aggressiveness of the disease and with patient outcome [11]. Functional studies suggest that the composition of the ECM is critical for HGS ovarian cancer cell growth and immune cell phenotypes [12], [13]. Hence, to study the biology of omental metastasis and develop screening platforms for personalised medicine, it is important that *in vitro* models contain an ECM similar to that found in patients. Most *in vitro* models developed so far use rat tail collagen I and murine Matrigel [14] as scaffold matrix [15], [16], [17]. These matrices are widely used and are excellent tools for *in vitro* and *in vivo* studies. However, they are of non-human origin, and they do not recapitulate the composition of the tumour ECM in patients (i.e. collagen does not recapitulate the molecular complexity of the tumour ECM and Matrigel primarily consists of basement membrane proteins, such as laminin 111 and collagen IV, entactin and heparan sulfate proteoglycan [14]). Matrix scaffolds derived from decellularized human tissues offer an important step forward in recapitulating the tumour ECM [18]; however, they are rather challenging to generate. Of note, all those matrices have largely been used and optimised to study cancer cell functions, while we know much less on how they influence phenotype and functions of tumour stromal cells.

Here we have developed omentum gel (OmGel), a matrix derived from tumour-associated omental tissue of HGS ovarian cancer patients. OmGel, but not Matrigel, contains most Matrisome proteins found in HGS omental tumours. When used in 2D and 3D *in vitro* assays to assess cancer cell functions, OmGel performs as well as or better than Matrigel. Conversely, OmGel and Matrigel distinctly modulated the phenotype of stromal cells.

We propose OmGel as a clinically and physiologically relevant matrix to study the biology of cells in HGS omental metastasis.

## Results

### OmGel recapitulates features of human tumour omentum

**Fig. 1:**
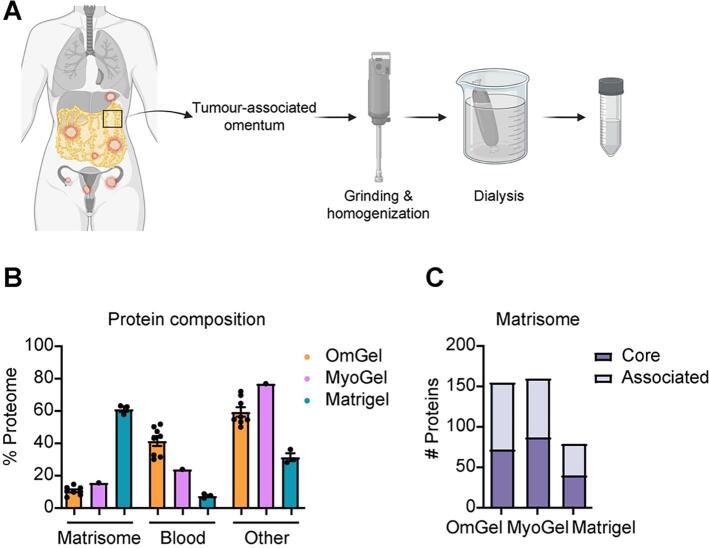

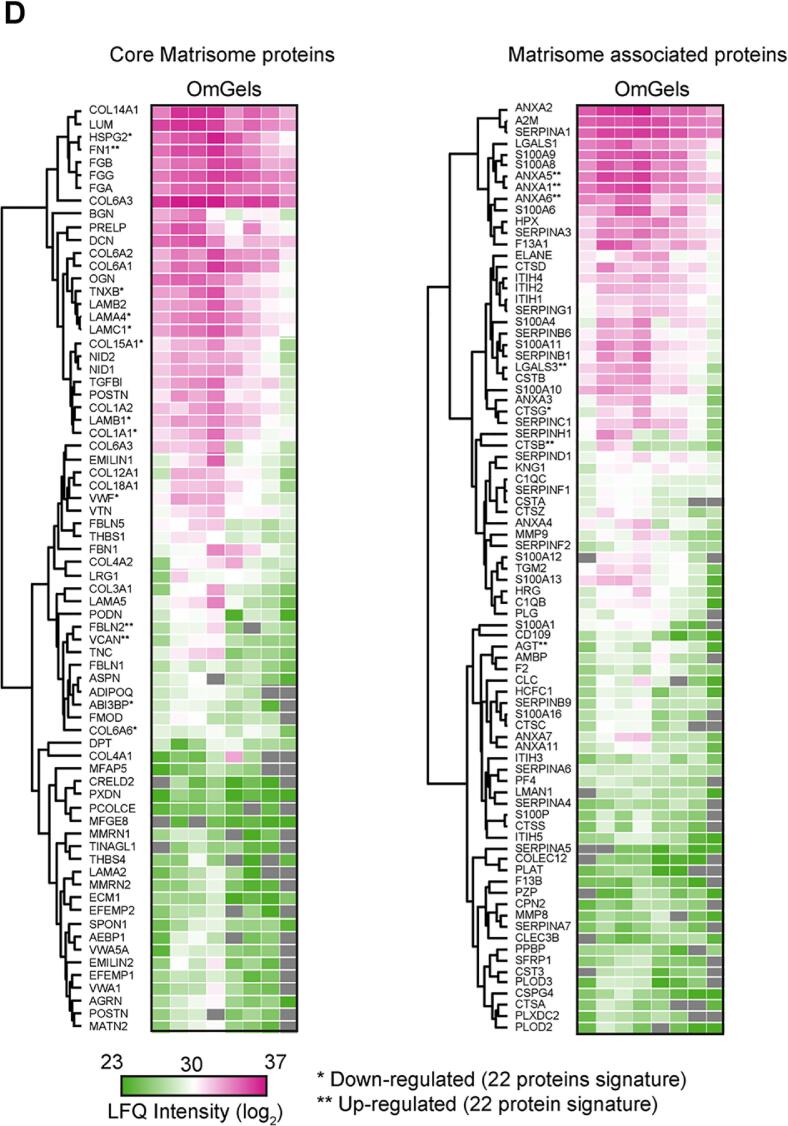
The OmGel contains a wide variety of matrisome proteins. A. Scheme of the OmGel preparation. Image made with Biorender. B. Estimated contribution (based on iBAQ intensities) ^54^ of Matrisome, blood and other proteins to the total proteome of OmGel, MyoGel and Matrigel. Each dot corresponds to a different sample (for OmGel, 6 are from omentum derived from distinct patients and two are from the pool of 5 or 7 different OmGels). C. Number of core Matrisome and Matrisome-associated proteins identified in OmGel (in 6 out of 8 analysed OmGels), MyoGel and Matrigel. D. Heat map based on protein Intensity values of core Matrisome and Matrisome-associated proteins quantified in OmGels (in at least 6 out of 8 analysed OmGels). Hierarchical clustering based on Euclidean distance and heat maps were generated with the Perseus software ^51^. Proteins highlighted with * and ** are either upregulated or downregulated in the Matrisome signature associated with shorter overall survival described by Pearce et al ^11^.

We have previously developed a protocol to generate a matrix gel from human uterus benign leiomyoma tumour tissue (MyoGel [19]), based on the method described to prepare murine Engelbreth-Holm-Swarm (EHS) [20] sarcoma-derived matrix (e.g. Matrigel). Here, we optimised the method to generate OmGel from tumour-associated omental tissue obtained from debulking surgery of HGS ovarian cancer patients with metastatic disease that had received neoadjuvant chemotherapy (Fig. 1**A**). To determine OmGel protein composition, and to assess interpatient sample variability and reproducibility in the OmGel preparation, we performed mass spectrometry (MS)-based proteomic analysis of six OmGels, each made independently from six patients. We additionally analysed two samples that were generated by mixing OmGels from 5 or 7 different patients (Table S1). MS analysis quantified 2,012 proteins in at least 75 % of the OmGel samples (6 of 8) and the average correlation of their abundance between samples was as high as 0.80 (min 0.72 – max 0.90, Figure S1). For comparison, we analysed the proteome of a MyoGel sample, as it is also of human origin but from different tissue [19] (Table S1). The average correlation between MyoGel and OmGel was lower (0.62, Figure S1**A**) than between OmGels, indicating distinct molecular composition of gels generated from different human tissues. Together this data indicates that the preparation of the OmGel is reproducible, as previously shown for MyoGels [19], and that the composition of OmGel derived from different patients is highly similar, consistent with the fact that all the omentum tissues were taken from patients with advanced HGS ovarian cancer.

Next, because of the active roles played by ECM proteins in regulating cell functions and tumour development, we investigated the presence of Matrisome [5] proteins in OmGel, MyoGel and Matrigel.

We estimated that Matrisome proteins, both core and associated components, accounted for more than 10 % of the OmGel and Myogel (Table S1). Conversely, Matrigel was made predominantly (60 %) of ECM proteins (Table S2). Interestingly, manual inspection of the OmGel proteome highlighted blood proteins among the most abundant. Consistently, when we estimated blood protein content based on a previously published plasma proteome [21], we found that they accounted for 40 % of the OmGel proteome (Fig. 1**B** and Table S1). This result is in line with omentum being a highly vascularised tissue [2]. Conversely, blood proteins represented 20 % or less of the Myogel and Matrigel proteome (Fig. 1**B** and Table S1). Another striking difference between patient-derived gels and Matrigel was the variety of the Matrisome proteins. OmGel contained more than 150 Matrisome proteins, similar to MyoGel, while Matrigel fewer than 80 (Fig. 1**C, D** and Tables S1**,S2**). Most Matrisome proteins in the Matrigel were identified in both OmGel and MyoGel (94.3 %) and their abundance had only a low positive correlation (Pearson correlation around 0.2, Figure S1**B,C** and Table S3). Conversely, Matrigel and Myogel shared more than 60 % of Matrisome proteins and their abundance had a higher correlation (Pearson correlation around 0.5, Figure S1**B,C** and Table S3).

Hence, our proteomic analysis shows that OmGel recapitulates key molecular features of the tumour omental microenvironment. Next, we sought to assess molecular similarities between OmGel and HGS omental tumours.

### OmGel molecularly recapitulates the stroma of HGS omental metastasis

**Fig. 2:**
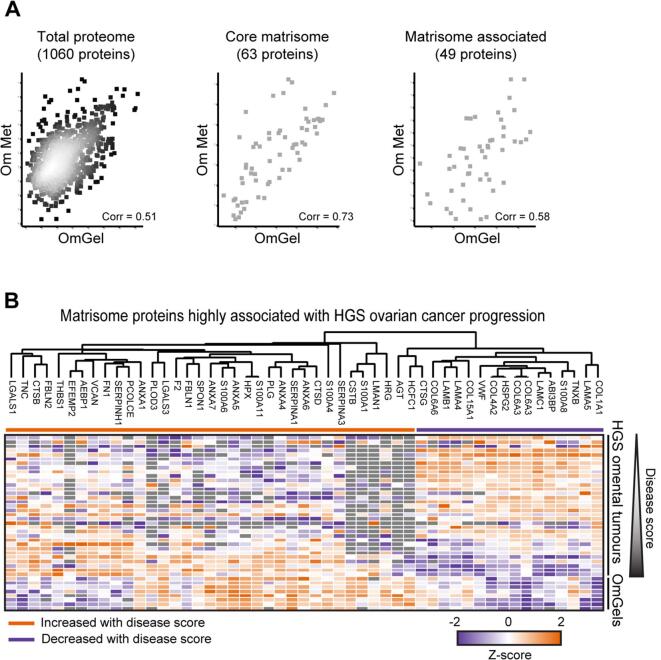

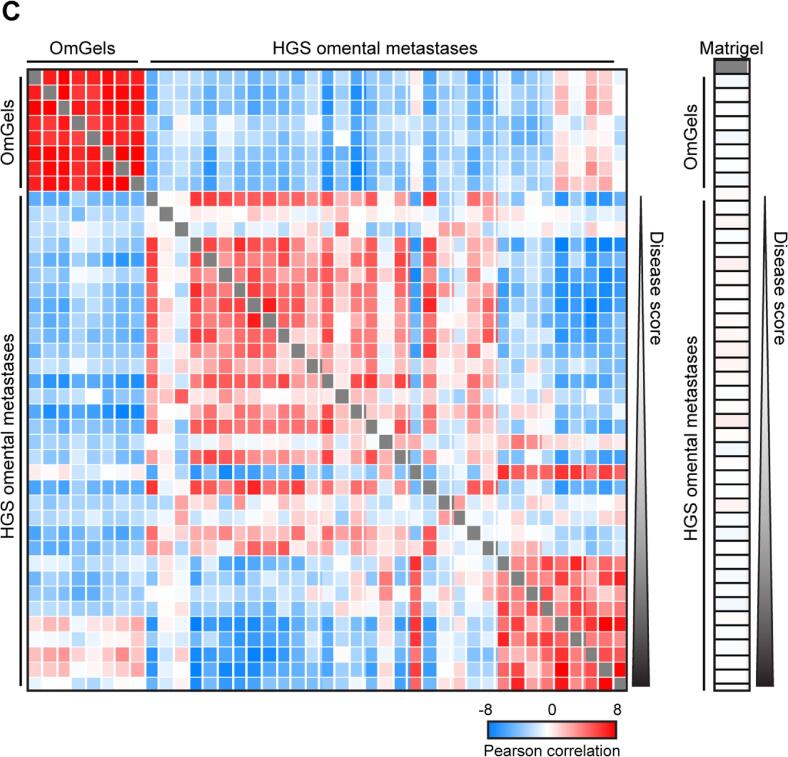
The OmGel proteome is similar to the proteome of ECM-enriched HGS omental metastasis. A. Dot plots of the average Intensities of proteins quantified in OmGel (x axis, average of 8 OmGels) and in ECM-enriched fraction of HGS omental tumour tissues (Om Met, y axis, average of 33 patient samples). From the left, plots show the total proteome, proteins of core Matrisome and proteins Matrisome associated. P = Pearson Correlation Coefficient. B. Heat Map based on normalised (by the median of each sample) Intensities measured by MS of a subset of proteins identified both in OmGel and ECM-enriched fraction of HGS omental tumour tissues, and that have been found co-regulated with disease score ^11^. C. Heat maps showing Pearson correlation coefficient of the 60 proteins that correlated with the disease score ^11^ between OmGel samples derived from different patients (A) and insoluble fraction of HGS omental metastasis tissues ^11^ (left) or Matrigel (right). Patient samples are ordered based on the disease score. The intensities of the proteins had been Z-scored prior to calculating the Pearson correlation. Heat maps were generated with the Perseus software ^51^.

Pearce et al. have previously identified a subset of Matrisome components that predicts the extent of HGS ovarian cancer disease (referred to as “disease score”) based on epithelial and stroma composition of the tumour tissue [11]. Among those, they identified a Matrisome gene expression signature associated with poor prognosis in HGS ovarian cancer and other cancer types [11]. Notably, this signature includes ECM proteins that can strongly influence tumour progression, such as collagen 1 (COL1A1) [7]. To assess whether OmGel is representative of potentially tumour promoting properties of HGS ovarian tumours, we compared our OmGel proteome with the proteome of the ECM-enriched fraction of HGS omental biopsies used to define the disease score. This cohort included 33 patients with disease that ranged from minimal to extensive [11]. We found 1,060 proteins identified both in the ECM-enriched fraction of HGS omental biopsies and in our OmGels (Table S4). Comparison of their normalised abundance, as measured by MS, showed an overall positive correlation of 0.51 (Pearson correlation coefficient). This correlation further increased to 0.73 when considering only the core Matrisome components (63 proteins), while remained similar, 0.58, when considering Matrisome-associated proteins (49 proteins) (Fig. 2**A** and Figure S1**D** for heatmap with quantified proteins). Among the top 60 Matrisome proteins that consistently increased or decreased with disease progression in HGS omental biopsies, we quantified 51 (85 %) in the OmGel, including COL1A1. The heat map in Fig. 2B highlights the strong similarity in abundance of most of those Matrisome proteins in OmGel and HGS omental biopsies. The correlation was particularly high with biopsies with high disease score, in line with our OmGels being derived from omentum of patients with advanced disease. Corroborating this observation, Matrisome protein levels in each of the OmGels positively correlated with the levels measured in HGS omental biopsies with highest disease score (Fig. 2**C** and Table S4). Conversely, only 15 (25 %) of the 60 proteins were identified in the Matrigel proteome and there was no correlation of their levels with those measured in the HGS omental biopsies (Fig. 2**C** and Table S4). Hence, the Matrisome of OmGel, but not of Matrigel, recapitulates that of advanced HGS omental tumours.

### OmGel can be used for cancer cell growth and invasion *in vitro* assays

The ability of ovarian cancer cells to aggregate into spheroids is important to metastasise to the peritoneal cavity from the primary site and to survive therapeutic treatments [22]. Moreover, the ability to move and invade is relevant to support further spread of the cancer cells within the metastasised organ. For these reasons, we sought to assess the utility of OmGel in *in vitro* assays to model the above processes and compared OmGel performance to that of the widely used Matrigel and of the MyoGel to assess the specificity of the OmGel.

**Fig. 3:**
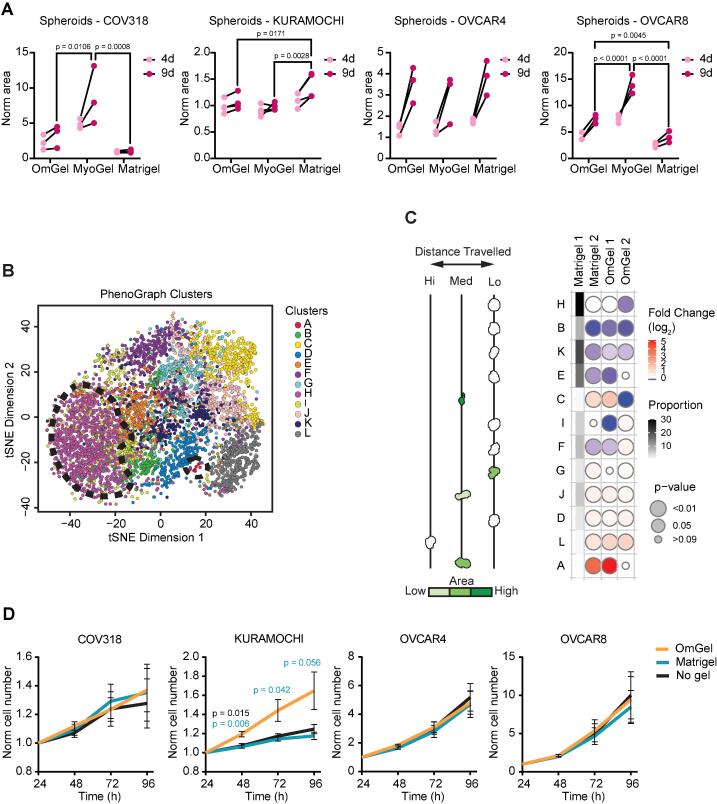
OmGel supports cancer cell motility and invasion. A. Plots showing the area of cancer cell spheroids grown for 4 days and 9 days (normalised to day 0) embedded in the indicated matrices. Each dot represents the average area of n = 6 spheroids. Black lines connect measurements performed at 4 and 9 days of the same biological replicate. N = 3 independent biological replicates. P-values calculated at 9 days based on 2-way ANOVA test corrected for multiple comparisons with Tukey’s test. B. t-SNE of states in CIOV5 spheroids grown on Matrigel and OmGel. Plot points colour denotes data-driven state classification. Black dashed lines highlight regions corresponding to data-driven states mentioned in text. Number of spheroids in experiment = 41,854, number of spheroids in subsampling = 20,000, iterations = 5,000, theta = 0.5, perplexity = 50. C. Quantification of data described in C. Representative spheroid shapes, arranged and coloured (light to dark green) based on average distance travelled by the cells over time and area occupied, respectively. Bubble heatmap, classification of spheroids as a log_2_ fold change from control (CIOV5 grown on Matrigel 1) (blue to red). Proportion of each class in the control sample Matrigel 1 is shown on grayscale heatmap (light = spheroid shapes found with low frequency). Bubble size represents p-values (bigger size = more significant), Cochran-Mantel-Haenszel test with Bonferroni-adjustment. n = 3 independent experiments, with 3 technical replicates per condition. D. Cancer cell growth, as measured by number of cells detected at the indicated time points according to Hoechst-stained nuclei. Cell numbers have been normalised to the number of cells measured 24 h after plating. P-values calculated between No gel vs OmGel or Matrigel using Kruskal-Wallis test corrected for multiple comparisons with Dunn’s test. Significant P-values were found when comparing No gel with OmGel for KURAMOCHI, at 48 h. N = 3 or 4 biological replicates. Error bars = SEM. (For interpretation of the references to colour in this figure legend, the reader is referred to the web version of this article.)

First, we performed a 3-Dimensional (3D) spheroid assay to measure cell aggregation and growth using four human ovarian cancer cell lines, of which KURAMOCHI, OVCAR4 and COV318 are highly representative of HGSOC [23], [24] and OVCAR8 is highly aggressive [25], [26]. The spheroid assay showed that, over nine days of growth, the four ovarian cancer cell lines embedded in OmGel formed spheroids of similar size compared to those grown in Matrigel, while COV318 and OVCAR8 spheroids grew bigger in MyoGel (Fig. 3**A** and Figure S2**A**).

Next, we assessed whether OmGel was suitable for *in vitro* experiments with patient-derived cells, and used the CIOV5 line, which has recently been established from the ascites of a HGS ovarian cancer patient [27]. We used an assay that we have previously established to assess the growth and invasive potential of cancer cell lines, in which single cells plated into Matrigel develop into 3D spheroids [28]. We used Traject3d, a method for label-free detection of 3D phenotypes in a cell culture [28] to determine phenotypic effects of the gels, comparing two different batches of OmGel and Matrigel. T-distributed stochastic neighbour embedding (tSNE) plots built using the repertoire of size, shape and movement features of spheroids grown in different gels, showed that cells developed into spheroids with a range of behaviours (Fig. 3**B**). These behaviours were found in both Matrigel and OmGel, and there were no batch effects (Figure S2**B,C**). The majority of behaviour states were similar between both OmGels, with the largest variation occurring between batches of Matrigel (Fig. 3**C**). Therefore, unbiased detection of spheroid behaviours revealed similar overall 3D phenotypes between OmGel and Matrigel. Hence, OmGel can be used to grow spheroids from patient-derived HGS ovarian cancer cells. Of note, we observed that patient-derived lines that did not grow well in Matrigel did not grow in OmGel either, suggesting that factors other than ECM and blood proteins are required for the growth of those cells.

Last, we assessed cancer cell growth when cultured in the presence (as coating of the plastic dish and in the medium) of OmGel, Matrigel or no gel for 96 h. For this, we used KURAMOCHI, OVCAR4, OVCAR8 or COV318 cell lines. Mirroring the lack of overt effect on 3D behaviours between OmGel and Matrigel, cancer cells grew similarly on all the matrices, as measured by cell number over time, except for KURAMOCHI, which grew faster in OmGel (Fig. 3**D**). Furthermore, MS proteomic analysis of the ovarian cancer cells cultured for 48 h in the presence of Matrigel or OmGel did not show any consistent difference across the different cell lines (Figure S3**A,** Table S5). This data collectively corroborates that OmGel is equivalent to Matrigel in supporting the culture of HGS ovarian cancer cells, and that OmGel does not have an additional phenotype or proteomic effect on the cells over and above Matrigel.

### OmGel influences CAF phenotypes

Given the unexpected finding that OmGel did not induce additional phenotypes to HGS cancer cells in culture, we turned our attention to other key components of the tumour microenvironment. In particular, cancer associated fibroblasts (CAFs). CAFs are a highly abundant cell type in the stroma of HGS omental tumours [11] and active regulators of ovarian cancer pathology [1], [29]. Moreover, CAFs are very plastic cells, and their phenotype and function can be determined by soluble factors [30] and by the structure and composition of self-produced ECM [31]. The plethora of single cell RNA sequencing (scRNAseq) data of tumours has unravelled several CAF phenotypes, of which myofibroblast-like CAFs (myCAFs), which produce abundant ECM, and inflammatory CAFs (iCAFs), which have enhanced expression of inflammatory markers, are found in most tumour types [32] and have been shown to be interconvertible [33].

To assess the effects of OmGel on CAFs, we used a line of patient-derived HGS omental CAFs expressing GFP (omCAFs) generated in our lab. We observed that omCAFs grown with OmGel or Matrigel or in the absence of a matrix acquired different phenotypes, which was in stark contrast with the cancer cells that did not show major changes when grown on different matrices (Fig. 3 and Figure S3**A**).

**Fig. 4:**
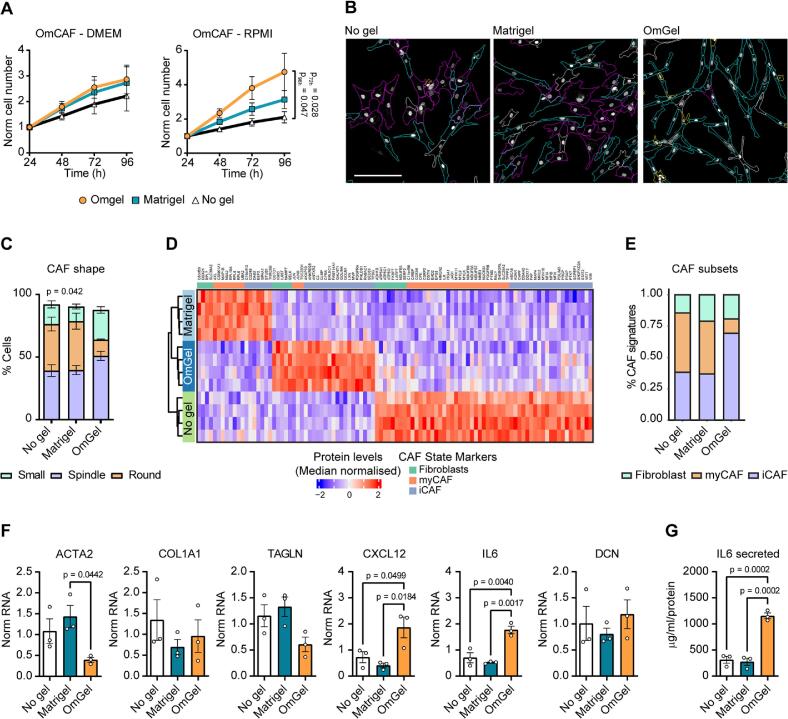
OmGel influences CAF phenotype and functions. A. CAF growth when cultured in DMEM or RPMI, as measured by number of cells detected at the indicated time points according to Hoechst-stained nuclei. Cell numbers have been normalised to the number of cells measured 24 h after plating. P-values calculated between No gel vs OmGel or Matrigel using Kruskal-Wallis test corrected for multiple comparisons with Dunn’s test. Significant P-values were found when comparing No gel with OmGel. N = 3 biological replicates. B, C. Representative images (B) and quantification (C) of CAF shapes when cultured for 48-h to reach 40–50 % confluency. Cell shapes were drawn using ImageJ program. Scale bar = 200 μm. D. Heat map and hierarchical clustering of differentially expressed proteins (one-way ANOVA FDR < 0.05) from CAFs grown with no gel, OmGel and Matrigel analysed by TMT MS proteomics. Protein level represents median normalised reporter ion intensities. N = 4 biological replicates. E. Summary of the proportion of CAF state markers that match with proteins upregulated in CAFs grown with no gel, OmGel and Matrigel. F. mRNA levels measured by qPCR of *ACTA2*, *COL1A1*, *TAGLN*, *CXCL12A*, *IL6* and *DCN* in omCAFs cultured with either OmGel, Matrigel or no gel for 48 h. P-values calculated with one-way ANOVA test comparing each experimental condition against each other. N = 3 independent biological replicates. G. IL6 protein levels measured by ELISA in the conditioned medium of omCAFs cultured with Omgel, Matrigel or no gel for 48 h. P-values calculated with one-way ANOVA test comparing each experimental condition against each other. N = 3 independent biological replicates.

First, omCAFs grew faster in the presence of OmGel than in the absence of a gel. This difference was statistically significant when cultured in RPMI and a trend when cultured in DMEM, as measured by changes in cell number over time (Fig. 4**A**). Conversely, Matrigel never induced a significant increase in CAF proliferation (Fig. 4**A**). Moreover, their shape had striking differences. OmCAFs grown in the absence of a gel displayed three distinct phenotypic shapes, small, round and spindle, with a similar frequency of spindle and round shape (Fig. 4**B,C** and Figure S3**C**). This was not significantly altered by the presence of Matrigel. However, in the presence of OmGel, omCAFs had predominantly a spindly, elongated shape (Fig. 4**B,C** and Figure S3**C**). Hence, this data reveals that unlike HGS ovarian cancer cells, OmGel promotes morphological alterations in omCAFs.

To measure in an unbiased manner how the different gels influenced CAF phenotype, we analysed their global proteome by MS. Proteomic analysis of omCAFs cultured for 48 h with OmGel, Matrigel or in the absence of gel identified significant differences between these conditions (Fig. 4**D**, Figure S3**D** and Table S6). To determine whether these differences were associated to known CAF phenotypes, we searched for enrichment of myCAF-, iCAF- and normal fibroblast-associated proteins, using fibroblast signatures determined in a recent pan-cancer scRNAseq study [34]. Among the regulated proteins, we found a large subset of proteins of the myCAF, iCAF and normal fibroblast signatures (Fig. 4**E.** Figure S3**D** and Table S7). CAFs grown with no gel upregulated the highest number of proteins and, similar to CAFs cultured with Matrigel, they upregulated both myCAF- and iCAF-associated proteins in similar proportion (Fig. 4**D,E** and Table S7). Strikingly, and in contrast to Matrigel, omCAFs cultured with OmGel upregulated mostly proteins of the iCAF signature (Fig. 4**D,E** and Table S7). Enrichment analysis for signatures associated to iCAF, myCAF and normal fibroblast further confirmed a significant enrichment for iCAF signature in omCAFs cultured with OmGel compared with Matrigel, and for both myCAF and iCAF signatures in omCAFs cultured in the absence of a gel (Figure S3**E**). To corroborate this observation, we measured mRNA levels of genes reported to be expressed in all CAFs (decorin, DCN) or highly expressed in iCAFs (C-X-C motif chemokine 12, CXCL12 and interleukin 6, IL6) and myCAFs (alpha smooth muscle actin, ACTA2, collagen 1, COL1A1 and transgelin, TAGLN [35], [36], [37]). OmCAFs cultured with OmGel had consistently higher CXCL12 and IL6 mRNA levels compared to Matrigel and no gel (Fig. 4**F**) and secreted significantly higher (4 folds) amounts of IL6 protein in the medium (Fig. 4**G**). Conversely, they had lower mRNA levels of the myCAF markers ACTA2 and TAGLN, although the latter did not reach statistical significance, compared to Matrigel (Fig. 4**F**). As expected, the levels of DCN mRNA were similar in all three culture conditions (Fig. 4**F**). Interestingly, these results mirrored the CAF phenotype analysis (Fig. 4**B,C**), suggesting that CAFs with a spindle shape are iCAF-like while CAFs with a round shape are myCAF-like, in line with common knowledge that myCAFs upregulate pathways involved in cell contractility [30]. This includes high expression of ACTA2, which we found upregulated in omCAFs cultured with Matrigel (Fig. 4**F**) and very abundant only in the omCAFs with a round shape, as assessed by immunofluorescence staining (Figure S3**F**).

Our data corroborates the knowledge that CAFs are highly plastic and that their phenotype is driven by tissue-derived factors, and the importance of using physiologically relevant matrices to study their biology.

## Discussion

Here we demonstrated the feasibility of using the omentum of patients with metastatic HGS ovarian cancer to produce a protein mixture with unprecedented similarity to the ECM of HGS omental tumours. OmGel is as simple as Matrigel to prepare, and we show that it can be used with both primary and commercial ovarian cancer cell lines in several *in vitro* assays. OmGel supports invasive behaviour of ovarian cancer cells and induces CAF phenotypes observed in tumours in patients, thus advancing the clinical and physiological relevance of models to study metastatic HGS ovarian cancer.

The omentum is a large flat adipose tissue layer that covers intra-peritoneal organs. Most patients with advanced HGS ovarian cancer undergo debulking surgery, during which the omentum is removed. While part of the resected omentum is used for pathological examination, most of it is left unused, and here we show that it can be exploited to generate a physiologically relevant matrix for *in vitro* studies. We found that OmGel has a molecular composition extraordinarily similar to that of the insoluble part of HGS omental metastasis, highlighting its clinical relevance. In addition to OmGel, cancer and other cell types can be extracted from the omentum of the same patient and used to build heterotypic ex-vivo models [15]. These models can be exploited for patient-specific functional studies, indicating the potential use of OmGel for personalised medicine.

Because the protocol to prepare OmGel includes a dialysis step, we expect it not to have large amounts of soluble factors, as also suggested by our proteomic analysis in which we could not consistently detect growth factors and cytokines. We can therefore speculate that the increased invasiveness of the cancer cells and changes in CAF phenotypes induced by OmGel had been driven by other factors, such as ECM proteins. However, other factors may have contributed to that, for example blood proteins like haemoglobin and albumin, which we have found highly abundant in the OmGel and which can influence cell functions [38].

Strikingly, our data unravel that when grown in normal culture conditions (in the absence of exogenously added gel) omCAFs have heterogeneous phenotypes, and that simply adding OmGel both to coat the culture dish and in the medium can drive CAFs toward a more inflammatory-like phenotype. Conversely, Matrigel or the absence of a gel upregulated many genes associated to myofibroblasts-like CAFs. We speculate that this is due to Matrigel containing higher content of ECM proteins than OmGel, which may enhance cell-ECM adhesion in omCAFs, which in turn triggers their myofibroblastic activation [31]. Furthermore, the absence of gel-coating may lead to higher stiffness of the dish, which has been shown to help to maintain myofibroblast-like phenotype in CAFs [39]. This may explain why there were fewer myCAF proteins upregulated in omCAFs cultured with OmGel. On the other hand, omentum tissue-derived factors contained in the OmGel may have triggered the upregulation of iCAF proteins. As there are limited protocols to grow iCAFs in culture and which require continuous inflammatory stimulation [33], [40], OmGel may provide a new tool to advance our understanding of iCAF biology. Further studies however are needed to determine the extent at which OmGel induces omCAFs towards an iCAF phenotype and the factors involved.

In conclusion, our work provides a step forward in recapitulating the omental metastatic environment to study the biology of HGS ovarian cancer metastasis. Metastasis in the omentum occurs in other cancer types, particularly gastric cancer, implying the relevance of OmGel to study the biology of other highly lethal cancers.

## Experimental procedures

### Patient samples and derived cells

Omental cancer associated fibroblasts (OmCAFs) or omentum gel (OmGel) were from the omentum of patients with an age range of 42 to 75 years obtained through NHS Greater Glasgow and Clyde Biorepository from tissues received from hospitals in the NHS Greater Glasgow and Clyde catchment area. All participants gave informed consent to use their tissue samples for research. The use of omentum tissue was approved by NHS GG&C Biorepository Management Committee at the Queen Elizabeth University Hospital, Glasgow. Patients were confirmed by a pathologist to have high grade serous ovarian cancer with metastatic spread to the omentum who have undergone debulking surgery post neo-adjuvant chemotherapy. The tissue used to prepare OmGel did not contain visible tumour, as assessed by a pathologist.

The CIOV5 cell line was originated from the HGS ovarian cancer patient-derived organoid PDO2 and has been previously characterised [27]. CIOV5 cells were maintained in DMEM/F12 supplemented with L-glutamine (Gibco) with 10 % FBS (Gibco) and 1 % penicillin–streptomycin (Gibco).

All cells were cultured at 37 °C, 5 % CO_2_, and tested negative for mycoplasma.

### OmGel preparation

Fresh omentum was washed briefly in cold phosphate buffered saline to remove excess surface blood, snap frozen in liquid nitrogen and kept at −80 °C until use. Omentum gel (OmGel) was prepared according to the methods described for the preparation of EHS sarcoma derived Matrigel (BD Biosciences [20]) and for Myogel [19] with minor modifications. Briefly, frozen omentum tissue was ground to a powder with a CryoMill (Retsch, Haan, Germany). Five grams of tissue powder was suspended in 5 ml of ice-cold urea buffer (2 M Urea, 0.05 M Tris, 0.154 M NaCl, pH 7.4) and homogenised using a T18 Ultra-Turrax (IKA®-Werke GmbH & Co. KG, Staufen, Germany). Homogenate was then processed as previously described [20]. The protein concentration in each preparation was measured using a DC Protein Assay (Bio-Rad) according to the manufacturer’s instructions with Victor3V 1420 Multilabel Counter (Perkin Elmer Life and Analytical Sciences, Turku, Finland). The OmGel solution was then stored in ≤ 0.5 ml aliquots at − 20 °C.

### Cancer cell culture

COV318 cells (Public Health England) were cultured in DMEM (Sigma-Aldrich or Gibco, Thermo Fisher Scientific) supplemented with 10 % fetal bovine serum (FBS, Gibco, Thermo Fisher Scientific), 100 U/ml penicillin, 100 µg/ml streptomycin (all from Sigma-Aldrich) and 2 mM glutamine (Gibco). KURAMOCHI (Tebu-Bio) and OVCAR4 (kindly provided by Prof. Charley Gourley, University of Edinburgh) and OVCAR8 (kindly provided by Prof. Kaisa Lehti, University of Helsinki) cells were cultured in RPMI (Gibco) supplemented with 10 % FBS, 2 mM glutamine (Gibco), 100 U/ml penicillin, 100 µg/ml streptomycin (all from Sigma-Aldrich). Cells were cultured at 37 °C, 5 % CO_2_ and tested negative for mycoplasma. Cell lines were authenticated using gDNA extracted from the cells using the Puregene Gentra Kit and multiplexed using the Promega Geneprint Kit. Samples were run on an Applied Biosystems 3130xl DNA analyser and analysed using Applied Biosystems Genemapper v4.1 software. Profiles were compared with ATCC (LGC standards) and both Cellosaurus and DSMZ databases.

### 3D tumour spheroid invasion assay

Spheroids were generated following the protocol described by Naakka et al. [41]. Briefly, the cells were seeded in 50 µl complete culture medium (see Cells and cell culture) at a cell density of 1 × 10^3^ cells/well into ultra-low attachment 96-well round bottom plates (Corning) and incubated at 37 °C for 4 days. After visual confirmation, spheroids were embedded in 50 µl gel containing 0.5 mg/ml Myogel [19] or OmGel, 0.5 mg/ml Fibrinogen (Merck), 0.3 U/ml Thrombin (Sigma-Aldrich) and 33.3 µg/ml of Aprotinin (Sigma-Aldrich) in complete culture medium of each cell line. For Matrigel (BD Biosciences) wells, the gel was diluted to 0.5 mg/ml with the complete culture medium of each cell line. The plate was then transferred to the incubator at 37 °C for 30 min to allow the gel to solidify. 100 µl of complete culture medium was added on top of the gels. Spheroids were imaged at 0 h and after 4 and 9 days of incubation at 37 °C, using a Nikon Eclipse TS100 inverted light microscope, with 4x objective magnification, connected to a Canon PowerShot S50 camera. Fiji software [42] was used for measuring the area covered by spheroids. The fold change in the total spheroid area at each time point compared with the area at 0 h was calculated.

### Live imaging and analysis of CIOV5 spheroids

CIOV5 cells were labelled with Green CMFDA CellTracker^TM^ (Invitrogen, C7025) diluted 1:1000 in normal medium for 30 min at standard conditions. Two different batches of Growth Factor Reduced Matrigel (GFRM) (Corning®, 354230) and two different batches of OmGel were used to coat the wells of a 96-well plate (ImageLock plate, Essen Biosciences) using 10 μl of each per well. Both Matrigel and OmGel coats were allowed to set at 37 °C for 15 min. Single cell resuspension of labelled CIOV5 cells was adjusted to a concentration of 1.5x10^4^ cells/ml, supplemented with 2 % Matrigel/ OmGel and added on top of the pre-coated wells (150 μl per well). Imaging was initiated following a 4-hour incubation at 37 °C using the IncuCyte® ZOOM system (Essen Biosciences) and a 10x objective lens. Phase and green channel images were obtained every hour for 3 days at 2 positions in the middle of each well.

### Phenotypic classification and trajectory identification

Data-driven phenotypic classification of the CIOV5 spheroids was performed using the Traject3D pipeline as described in [28], using an adapted version of the CellProfiler pipeline [43] (v4.2.0) and KNIME Analytics Platform [43] (https://www.knime.com/, v4.0.2) with R (https://cran.r-project.org/, v3.6.2) and Python (https://www.python.org/, v3.8) integrations. During imaging, the two OmGel batches tested were found to accumulate debris over time which was picked up on the Phase channel and made spheroid segmentation less accurate. To overcome this, some adaptations were implemented on the CellProfiler pipeline. Namely, we aimed to restrict the detection of spheroids within a limited area of each image in which we knew spheroids were found. To define the areas of detection, we labelled the spheroids with a green dye (CMFDA CellTracker^TM^), identified the areas with green signal within each frame and used those as a mask on the Phase image. The following modules and functions: *CorrectIlluminationCalculate*, *CorrectIlluminationApply*, (calculate and apply an illumination function to correct uneven illumination), *Colour to Gray* (turns a colour image to grayscale) and *RescaleIntensity* (rescales intensity range) were used on the green channel to define the green-positive areas. Those were identified (*EnhanceEdges* and *IdentifyPrimaryObjects*) and used as an enlarged mask on the Phase image (*MaskObjects* (mask the green spheroids), *ExpandOrShrinkObjects* (Expand the masked green spheroids by 3 pixels) and *MaskImage* (use the expanded green spheroids as a mask on Phase)). Once the Phase areas in which we expected to find spheroids were defined, spheroid detection was performed with the modules described before [28], with cell-line specific settings in each module to optimise segmentation.

The KNIME analytics platform was used as described before [28]. Briefly, the GeoScketch algorithm was used to subsample the CIOV5 data [44], and the PhenoGraph [45] algorithm with a *k*-nearest neighbours value of 40 was implemented to identify the spheroid states based on shape, size and movement measurements, but not temporal information. Trajectories were also identified by the PhenoGraph algorithm by reconstructing the temporal sequence of state classification and removing spheroids for which tracking was not available from the beginning of the experiment (cutoff at t = 20 h) or for which tracking was interrupted (more than 2 consecutive missing values). Quantitation for both states and trajectories is presented in bubble heatmap format generated using ggplot2 [46]. In each case, the proportion of each state/ trajectory in the control sample (Matrigel 1) is expressed in grayscale heatmap and the changes across conditions as log_2_ fold change from control sample. Statistical comparison was performed using the Cochran-Mantel-Haenszel test with Bonferroni adjustment, which allows for comparison to reach statistical significance only where the effect is observed across all experimental replicates. To test whether the magnitude of the effect was homogeneous across experimental replicates, we used the Woolf statistic (a non-significant p-value indicating a homogeneous effect and indicated with a black dot in the middle of the heatmap bubble). Row clustering was performed using the complete linkage method for hierarchical clustering of the Euclidian distances. The trajectory motifs were generated using the ggseqlogo R package [47], where state proportion in 12 h intervals is indicated by symbol (letter) height. t-SNE mapping of cell states was performed using the Rt-SNE package (https://github.com/jkrijthe/Rtsne) as described before [28]. Similarly, representative outlines of each spheroid state were generated as in [28].

### Omental CAF cell line generation and culture

Fresh omentum from a 66 year old patient diagnosed with ovarian high grade serous carcinoma was finely dissected and put in pre-warmed and filtered hyaluronidase (250 U/ml, Sigma) and collagenase (2 mg/ml, Roche) in DMEM (Gibco, Thermo Fisher Scientific) on a rotator at 37 °C overnight. The digested tissue was then filtered through a cell strainer and centrifuged. The CAF-containing cell pellet was washed in PBS and resuspended in DMEM supplemented with 10 % FBS (Gibco, Thermo Fisher Scientific), 1 % penicillin/streptomycin (Life technologies) and 2 mM glutamine (Gibco, Thermo Fisher Scientific). Patient-derived omental CAFs (omCAFs) were obtained by sorting of the mixed cell population using flow cytometry. Cells were detached using Accumax (Thermo). Fluorescently conjugated antibodies (APC-CD90, FITC-CD31, FITC-CD45, PE-EpCAM all from Biolegend) were added to the cell suspension (2 μl per 1 million cells) and incubated on ice for 30 min with mixing every 10 min. Cells were washed and also resuspended in 10 ml of FACS sorting buffer (PBS, 3 % FBS, 25 mM HEPES, 5 mM EDTA) filtered through a 70 μm cell strainer and resuspended in FACS sorting buffer at a concentration of 1 million cells/ml. Cells were sorted on a BD fusion sorter alongside the appropriate staining and FMO controls utilising OVCAR8 and HUVEC cells prepared as above as positive controls for EpCAM and CD31, respectively. CAFs were defined as FITC negative, PE negative and APC strongly positive cells. Sorted CAFs were then immortalised using a human telomerase reverse transcriptase (hTERT)-expressing plasmid (pIRES2-hygro, kindly provided by Ferdinando Calvo, Instituto de Biomedicina y Biotecnología de Cantabria). Lentivirus containing the hTERT plasmid was generated in HEK293T cells. Two rounds of viral transduction were performed on consecutive days. CAFs were selected using hygromycin (10 µg/ml; Sigma).

### Cell proliferation with Opera Phenix

4.5x10^3^ immortalised GFP expressing omCAFs or 4.5x10^3^ KURAMOCHI or COV318 or 0.9x10^3^ OVCAR4 or OVCAR8 HGS ovarian cancer cells labelled with 1 µM CMPTX cell tracker red (Invitrogen) (both omCAF and cancer cell nuclei were labelled with 20 µM Hoechst) were plated per well of a 96 well PhenoPlate (Perkin Elmer) in either RPMI (KURAMOCHI, OVCAR4, OVCAR8) or DMEM (COV318) containing 30 µg/ml OmGel (mix of OmGels derived from 6 HGS ovarian cancer patients) or Matrigel (growth factor reduced, BD Biosciences) supplemented with 4 % FBS, 1 % penicillin/streptomycin and 1 % glutamine (all from Life Technologies). Cells were plated on plastic wells not coated or pre-coated with either 30 µg/ml OmGel or growth factor reduced Matrigel. Images were taken on an Opera Phenix high-throughput confocal microscope (Perkin Elmer) at 37 °C and 5 % CO_2_. Nine images were taken per well using 10x objective with sequential excitation at 405 nm, 488 nm, 561 nm. Images were taken every 24 hrs over a 96 hr period. To quantify cell number, analysis was done using Harmony 4.9 Software (Perkin Elmer) to segment nuclei and cells. Supervised machine learning using Harmony’s “Phenologic” function, allowed cells to be classified into three observed, morphological categories.

### ELISA

Conditioned medium was collected from 4.5x10^3^ omCAFs grown per well on a 96 well plate coated with 30 µg/ml of either Matrigel or OmGel or uncoated for 48 h in RPMI supplemented with 4 % FBS, 1 % penicillin/streptomycin, 2 mM glutamine. The medium was centrifuged at 4 °C for 5 min at 5,000 g and the supernatant was collected. To normalise for differences in cell number, relative cell numbers were quantified using a methylene blue assay. Briefly, the plates were washed with warmed PBS then fixed with 10 % normal buffered saline (NBF) for 10 min at 37 °C. After a repeat wash with PBS, the plates were stained with 1 % methylene blue in 10 mM borate buffer/50 % methanol pH 8.5 for 30 min at room temperature. The plates were washed with distilled water and destained using 50:50 (vol:vol) ethanol and 0.1 M HCl. Absorption was measured at 630 nm using a SpectraMax ABS plus (Molecular Devices). The ELISA analysis was performed using the Human IL6 uncoated ELISA kit (Invitrogen) as per the manufacturer’s instructions and read at 450 nm. Result was divided by the methylene blue assay 630 nm absorption values.

### Immunofluorescence staining

2x10^4^ omCAFs were grown per well of a 24 well plate on a glass coverslip either coated with 30 µg/ml of Matrigel or OmGel or uncoated for 48 h in RPMI supplemented with 4 % FBS, 1 % penicillin/streptomycin, 2 mM glutamine. After two PBS washes, cells were fixed in 10 % neutral buffered formalin (Sigma-Aldrich) for 20 min. Formalin was removed and after a further two PBS washes, 1 % BSA/0.1 % saponin (both Sigma) in PBS was applied to block and permeabilise the cells for 30 min at room temperature. Cells were incubated overnight at 4 °C in 1:1000 mouse monoclonal anti-alpha smooth muscle actin antibody (Abcam, ab7817) in 1 % BSA/0.1 % saponin in PBS. Cells were washed three times in 1 % BSA in PBS prior to application of donkey anti mouse Alexa fluor 555 (Life Technologies, A31570) secondary antibody at 1:500 in 1 % BSA/0.1 % saponin in PBS for 1 h RT. Following a wash in 1 % BSA in PBS, Dapi (Abcam ab228549) at 1:2000 in 1 % BSA in PBS was applied for 20 min at room temperature. After a final wash in 1 % BSA in PBS, the coverslips were mounted onto glass slides using fluorescent mountant (Dako). Images were captured using a 40x objective on a Zeiss 710 confocal microscope.

### Reverse transcription polymerase chain reaction (RT-qPCR)

DNase treatment and total RNA isolation were performed using the RNeasy mini kit (Qiagen, Hilden, Germany) according to the manufacturer’s instructions. 1 μg of RNA was used to synthesize complementary DNA using the iScript kit (BioRad). DNA was diluted to 10 ng/μl and 2 μl were used in each RT-qPCR reaction with 10 μl of iTaq Universal SYBR Green Supermix (Bio-Rad Laboratories, Hercules, CA) and 400 nM of forward and reverse primers. PCR runs were performed using a QuantStudio^TM^ 3 Real-Time PCR System (Thermo Fisher Scientific). The following primers were used: ACTA: GTGTGCCCCTGAAGAGCAT (Fw) GCTGGGACATTGAAAGTCTCA (Rev); TAGLN1: GGTGGAGTGGATCATCGTGC (Fw) ATGTCAGTCTTGATGACCCCA (Rev); COL1A1: TGAAGGGACACAGAGGTTTCAG (Fw) GTAGCACCATCATTTCCACGA (Rev); IL6: GGTACATCCTCGACGGCATCT (Fw) GTGCCTCTTTGCTGCTTTCAC (Rev); CXCL12: CTACAGATGCCCATGCCGAT (Fw) CAGCCGGGCTACAATCTGAA (Rev); 18S: AGGAATTGACGGAAGGGCAC (Fw) GGACATCTAAGGGCATCACA (Rev); DECORIN: GGGCTGGCAGAGCATAAGTA (Fw) CAGAGCGCACGTAGACAT (Rev).

### Sample preparation for MS proteomic analysis

3x10^5^ omCAFs or HGS ovarian cancer cells were plated on 10 cm dishes not coated or coated with 30 µg/ml Matrigel or OmGel (mix of OmGels derived from 6 HGS ovarian cancer patients) and grown for 48 h in RPMI supplemented with 4 % FBS, 1 % penicillin/streptomycin, 2 mM glutamine with or without 30 µg/ml OmGel or Matrigel. Cells were washed 3 times in PBS then lysed in lysis buffer containing 4 % SDS, 0.1 M dithiothreitol (DTT, Sigma) in 0.1 M Tris-HCl pH7.4. Proteins were precipitated overnight at −20 °C in cold acetone, resuspended in 50 mM HEPES (Sigma) pH 8.0, alkylated in 55 mM iodoacetamide (IAA, Sigma) in the dark for 45 min at room temperature and digested with Lys C (Wako chemicals) for 3 h at RT and then with trypsin (Promega) overnight at 37 °C. For the CAF phenotype experiment, peptides were labelled with TMTpro 16plex reagent kit according to manufacturer instructions (Thermo Scientific). An aliquot of of each sample was mixed and desalted using C18 StageTip [48] prior to an incorporation test. The sum of the ion intensities was used to determine quantity of each sample that was pooled and then fractionated (see below). For all other experiments, peptides were acidified with trifluoracetic acid (TFA, Sigma) and desalted using C18 StageTip [48] prior to MS analysis. Peptides were eluted with two rounds of 20 µl of 80 % acetonitrile (ACN, VWR) 0.1 % formic acid (Millipore).

For the gels, OmGel and Matrigel samples were resuspended in lysis buffer and prepared as described above.

### High pH offline fractionation of TMT-labelled peptides

800 µg of mixed TMT-labelled peptides were fractionated on an Agilent 1260 Infinity II HPLC. Solvent A (2 % ACN, 98 % water) and B (90 % ACN, 10 % water) were adjusted to pH 10 using ammonium hydroxide. Samples were manually injected using a rheodyne valve and subjected to a two-step gradient, 2–28 % solvent B in 39 min then 28–46 % solvent B in 13 min. The column was washed for 8 min with 100 % Solvent B followed by a re-equilibration for 7 mins. The flow rate was set to 200 µl/min and samples collected into 21 fractions, which were run separately at the MS.

### MS analysis

Digested peptides were run on the Thermo Scientific Orbitrap Lumos mass spectrometer (Thermo Scientific) coupled to an EASY-nLC II 1200 chromatography system (Thermo Scientific). Samples were loaded on a 50 cm fused silica emitter packed in-house with ReproSIL-Pur C18-AQ, 1.9 µm resin (Dr. Maisch) heated to 55 °C using a column oven (Sonation).

For the unlabelled samples, peptides were eluted at a flow rate of 300 nl/min over a 275 min two-step gradient method. The % of solvent B (80 % ACN, 0.1 % FA) was 2–22 % at step one (180 min) and 32 % (50 min) at step two. For the TMT-labelled samples, peptides were eluted at a flow rate of 300 nl/min over three optimised two-step gradient methods for fractions 1–7, 8–15 and 16–21. Step one was commenced for 75 mins and step two for 25 min. For fractionated samples 1–7 the % of solvent B was 3–18 % at step one and 30 % at step two. For fractions 8–15 the % of B was 5–24 % at step one and 38 % at step two and for fractions 16–21 the % B was from 7 to 30 % at step one and 47 % at step two. Peptides were then electrosprayed into the mass spectrometer using a nanoelectropsray ion source (Thermo Scientific). An Active Background Ion Reduction Device (ABIRD, ESI Solutions) was used to decrease air contaminants. Data was acquired using Xcalibur software (Thermo Scientific) in positive mode using data-dependent acquisition. Full scan mass range was set to 375–1500 *m*/*z* at 120,000 resolution. For the unlabelled peptides, injection time was set to 50 ms and high collision dissociation (HCD) fragmentation triggered on the top 20 most intense ions. For MS2 analysis, injection time was set to 20 ms and ions were detected in the ion trap with *m*/*z* isolation set to 1.4. Dynamic exclusion was set to 45 s. For the TMT-labelled peptides, injection time was set to 50 ms with a target value of 5E5 ions. HCD fragmentation was triggered at top speed (3 s) for MS2 analysis. MS2 injection time was set to 175 ms with a target of 2E5 ions and 15,000 resolution with *m*/*z* isolation set to 0.8. Ions already selected for MS2 were dynamically excluded for 30 s.

### MS data analysis

MS.raw data was processed with MaxQuant software [49], version 1.6.3.3 for the gels and 1.6.14.0 for the cancer cell and CAF proteomes, and searched with the Andromeda search engine [50] against the Uniprot *Homo Sapiens* database (2018, 95,146 entries). For TMT-labelled samples, data was searched with multiplicity set to MS2 level TMT16plex. For unlabelled samples, LFQ and iBAQ quantification options were enabled. First and main searches were done with a precursor mass tolerance of 20 ppm for the first search and 4.5 ppm for the main. MS/MS mass tolerance was set to 20 ppm. Minimum peptide length was set to 7 amino acids and trypsin cleavage was selected allowing up to 2 missed cleavages. Methionine oxidation and N-terminal acetylation were selected as variable modifications and Carbamidomethylation as a fixed modification. FDR was set to 1 % for both protein and peptide identification.

### MS data analysis of OmGel, Matrigel and myogel proteome

The MaxQuant output proteinGroups file was processed using Perseus software [51] version 1.6.15.0. Contaminants, reverse identification and proteins identified only by site were removed, and only proteins identified with at least 1 unique peptide were kept for the analysis. For the OmGel proteomes, only proteins quantified in six of the eight samples were used for downstream analysis. For correlation analysis between OmGel samples Pearson correlation coefficient was calculated based on Intensity values calculated by MaxQuant.

### MS data analysis of cancer cell grown on Matrigel and OmGel

Analysis was performed using R version 4.2.2 [52]. For the cancer cell comparison, LFQ protein intensities were filtered to remove contaminants, only identified by site, identified in reverse, less than 1 unique peptide and proteins with greater than 80 % valid values. Missing values were imputed using the K-nearest neighbour method of the Impute R package. LFQ intensities were log transformed and median normalised. Differential expression analysis was performed using the DEqMS statistical method [53]. Volcano plots were created using log_2_FC and p.value and significantly differentially expressed proteins were defined as absolute log_2_FC > 0.5 and FDR < 0.05.

### Comparison MS proteomic data with insoluble proteome of HGSOC omental tumours

Matrisome proteins, including Core and Associated proteins, were defined according to the in silico Human Matrisome (https://matrisomeproject.mit.edu/). Blood proteins were defined according to the plasma proteome in [21]. iBAQ intensities [54] were used for the proteomes of the gels. The proteomic data of the insoluble fraction of HGS omental tumours was from [11]. To compare the proteome of the gels with that of the insoluble part of the HGS omental tumours, for each sample, the intensity values of each protein, either calculated by MaxQuant (Intensity Based Absolute Quantification, iBAQ, for the gels) and those reported in Pearce et al. [11], were normalised by the median intensity value of all the proteins in the sample. Heat maps, dot plots and calculation of Pearson correlation, and hierarchical clustering were performed with Perseus software [51].

### Downstream analysis of CAF phenotypes

The proteinGroups file from MaxQuant was used for downstream analysis of CAF phenotypes using R version 4.2.2 [52]. Proteins were filtered to remove contaminants, only identified by site, identified in reverse, less than 1 unique peptide and proteins with at least one missing value. To minimise the contribution of the OmGel proteins, plasma proteins [21] and Matrisome proteins [5] were removed. Corrected reporter ion intensity were normalised by the median intensity. One-way analysis of variance (ANOVA) test was performed and a heatmap of differentially expressed proteins (FDR < 0.05) was produced using the ComplexHeatmap package [55]. Hierarchical clustering was performed using Euclidean distance and k-means clustering of rows into six groups. Condition specific clusters were extracted to identify upregulated proteins unique to CAFs grown on no gel, Matrigel and OmGel. CAF state signatures defined by Luo et al. [34] were filtered to remove marker genes present in more than one CAF state. Upregulated proteins unique to CAFs grown on no gel, OmGel and Matrigel were mapped to CAF state signatures and the proportion of matches for each state were calculated. For direct comparison between conditions, differential expression analysis was performed using the DEqMS. This method considers the variance in peptide counts and is more accurate in detecting differentially regulated proteins at the same FDR compared to other statistical methods [53]. Proteins were ranked based on log fold-change and gene-set enrichment analysis was performed using the fgsea package (https://doi.org/10.1101/060012). Results of the DEqMS analysis between OmGel and Matrigel were also used to create a volcano plot of log_2_FC and p-value. Significantly differentially regulated proteins were defined as absolute Log_2_FC > 0.5 and FDR < 0.05.

### Statistical analysis

GraphPad Prism v.6.0 was used for statistical analysis. Statistical tests are reported in the figure legend. A p-value of ≤ 0.05 was considered significant. All graphs show the mean ± SEM of at least three biological replicates (independent experiments) unless otherwise stated.

### CRediT authorship contribution statement

**Lisa J. Neilson:** Methodology, Investigation. **Douglas Cartwright:** Methodology, Investigation, Writing – review & editing. **Maija Risteli:** Methodology, Investigation. **Elina M. Jokinen:** Investigation. **Lynn McGarry:** Methodology, Investigation. **Toni Sandvik:** Investigation. **Konstantina Nikolatou:** Methodology, Investigation. **Kelly Hodge:** Methodology, Investigation. **Samuel Atkinson:** Investigation. **Maria Vias:** Resources. **Emily J. Kay:** Investigation. **James D. Brenton:** Resources. **Leo M. Carlin:** Methodology, Supervision. **David M. Bryant:** Methodology, Investigation, Supervision. **Tuula Salo:** Investigation, Supervision. **Sara Zanivan:** Methodology, Investigation, Supervision, Writing – original draft.

## Declaration of Competing Interest

The authors declare that they have no known competing financial interests or personal relationships that could have appeared to influence the work reported in this paper.

## Data Availability

The raw MS files and search/identification files obtained with MaxQuant have been deposited to the ProteomeXchange Consortium (https://proteomecentral.proteomexchange.org/cgi/GetDataset) via the PRIDE partner repository [56] with dataset identifier PXD045024 for Matrigel proteome, PXD042317 for TMT experiment and dataset identifier PXD042320 for all the other label-free experiments. All unique materials used are readily available from the authors.
