# Supplementary material for: Omentum-derived matrix enables the study of metastatic ovarian cancer and stromal cell functions in a physiologically relevant environment
Source: Matrix Biol Plus. 2023 Nov 22;19-20:100136. doi: 10.1016/j.mbplus.2023.100136 (PMC10784634; doi:10.1016/j.mbplus.2023.100136)
Supplement: Supplementary data 1 [file mmc1.docx]

**Supplementary Figures and Tables**

**Figure S1. Correlation of the proteome of OmGels derived from different patients**

**A**. Scatter plots showing Pearson correlation coefficient of the protein Intensities of the proteome of OmGels derived from different patients (OmGel 1-5), of OmGels derived from 5 and 7 different patients that were pooled together (OmGel Mix 1 and 2), and of a MyoGel. Only proteins identified in at least 75% of the OmGel samples (6/8) were used for this analysis. Heat maps were generated with the Perseus software ^51^. **B**. Venn diagram of the Matrisome proteins identified by MS analysis in the three different matrices (Matrisome proteins quantified in 6 out of 8 OmGels, 2 out of 3 Matrigels and 1 Myogel). **C.** Heat Map based on normalised (by the median of each sample) Intensities measured by MS of the Matrisome proteins identified in the OmGels and for which the corresponding protein was quantified in Myogel (Myo) or Matrigel. Grey means that the protein was not quantified in the sample by MS analysis. **D.** Heat Maps based on normalised (by the median of each sample) Intensities measured by MS of core matrisome (left) and matrisome associated (right) proteins identified both in OmGel and ECM-enriched fraction of HGS omental tumour tissues. HGS omental tumour are ordered based on their disease score (highest on the right) as defined in Pearce et al. ^11^. Grey means that the protein was not quantified in the sample by MS analysis.

**Figure S2.** **A.** Representative images of the spheroids quantified in Figure 3A. Scale bar = 500 µm. Images were acquired on a Nikon Eclipse TS100 inverted light microscope, with 4x objective magnification, connected to a Canon PowerShot S50 camera, and converted to greyscale after acquisition. **B.** t-SNE of spheroids in CIOV5 spheroids grown on Matrigel and OmGel. Plot points colour denotes sample of origin for each spheroid. Experimental details and tSNE parameters described in Figure 3C,D. **C.** Bubble heatmap, quantitation of trajectory classification, proportion of each trajectory in reference samples is shown on grayscale heatmap, log_2_ fold change from control (CIOV5 grown on Matrigel 1) (blue to red). Bubble size represents p-values, Cochran-Mantel-Haenszel test with Bonferroni-adjustment. Black dot represents p-value, Woolf test Bonferroni-adjusted, for homogeneity of odds ratio across experiments. Experimental details described in Figure 3C,D.

**Figure S3. OmGel and Matrigel distinctly influence the proteome of CAFs but not of cancer cells.**

**A.** Volcano plot of protein levels, as measured by MaxQuant LFQ, comparing cancer cells grown with Matrigel and OmGel using DEqMS statistical test. Results from 4 cells lines (COV318, Kuramochi, OVCAR4, and OVCAR8) were combined with N = 2-3 biological replicates per cell line. Dotted lines represent DEqMS log_2_FC +/- 0.05 and Benjamini-Hochberg corrected FDR < 0.05. **B.** Volcano plot of protein levels, measured by TMT MS, comparing CAFs grown on Matrigel and OmGel using DEqMS statistical test. N = 4 biological replicates. Dotted line represents log_2_FC +/- 0.5 and Benjamini-Hochberg corrected FDR < 0.05. **C.** Representative confocal microscope images used for cell shape analysis of GFP-expressing omCAF cultured with the different gels for 48 h. The regions highlighted with a dashed line are those shown in Figure 4B, which were used as example of cell shape annotation. Images were acquired on a Opera Phenix high-throughput confocal microscope (Perkin Elmer) using an objective with 10x magnification. Scale bar = 50 µm. **D.** Heatmap of proteins upregulated in CAFs grown with no gel, OmGel and Matrigel that are present in CAF state signatures ^34^. Columns clustered using Euclidean distance. **E.** Normalised enrichment score calculated using gene set enrichment analysis of CAF states signatures. Proteins were ranked by log fold change (differential protein analysis using DEqMS). **F.** Representative images of αSMA staining in omCAFs grown with different matrices for 48 hours. Images were acquired on a Zeiss 710 confocal microscope using an objective with 40x magnification. Scale bar = 50 µm.

**Table S1. Proteome of OmGel and MyoGel**

**Table S2. Proteome of Matrigel**

**Table S3. OmGel Matrisome compared to Myogel and Matrigel Matrisome**

**Table S4. Normalised proteome of OmGels and ECM enriched HGS omental tumours**

**Table S5. Proteome of cancer cell lines grown with Matrigel or OmGel**

**Table S6. Proteome of omCAFs grown with Matrigel or OmGel or with no gel**

**Table S7. Regulated proteins in omCAFs grown with OmGel, Matrigel and no gel from fibroblast subsets in pan cancer study**
